# Genome-wide analysis of TALE superfamily in *Triticum aestivum* reveals *TaKNOX11-A* is involved in abiotic stress response

**DOI:** 10.1186/s12864-022-08324-y

**Published:** 2022-01-31

**Authors:** Yuxuan Han, Lili Zhang, Luyu Yan, Xinxin Xiong, Wenjing Wang, Xiao-Hong Zhang, Dong-Hong Min

**Affiliations:** 1 State Key Laboratory of Crop Stress Biology for Arid Areas and College of Agronomy, Northwest A&F University, Yangling, Shaanxi China; 2 State Key Laboratory of Crop Stress Biology for Arid Areas and College of Life Sciences, Northwest A&F University, Yangling, Shaanxi China; 3 Shaanxi Agricultural Machinery Appraisal and Extension Station, Xian, Shaanxi China

**Keywords:** TALE, KNOX, BELL, Genome-wide analysis, Abiotic stresses, Wheat

## Abstract

**Background:**

Three-amino-loop-extension (TALE) superfamily genes are widely present in plants and function directly in plant growth and development and abiotic stress response. Although *TALE* genes have been studied in many plant species, members of the TALE family have not been identified in wheat.

**Results:**

In this study, we identified 70 wheat TALE protein candidate genes divided into two subfamilies, KNOX (KNOTTED-like homeodomain) and BEL1-like (BLH/BELL homeodomain). Genes in the same subfamily or branch in the phylogenetic tree are similar in structure, and their encoded proteins have similar motifs and conserved structures. Wheat *TALE* genes are unevenly distributed on 21 chromosomes and expanded on the fourth chromosome. Through gene duplication analysis, 53 pairs of wheat *TALE* genes were determined to result from segmental duplication events, and five pairs were caused by tandem duplication events. The Ka/Ks between *TALE* gene pairs indicates a strong purification and selection effect. There are multiple cis-elements in the 2000 bp promoter sequence that respond to hormones and abiotic stress, indicating that most wheat *TALE* genes are involved in the growth, development, and stress response of wheat. We also studied the expression profiles of wheat *TALE* genes in different developmental stages and tissues and under different stress treatments. We detected the expression levels of four *TALE* genes by qRT-PCR, and selected *TaKNOX11-A* for further downstream analysis. *TaKNOX11-A* enhanced the drought and salt tolerances of *Arabidopsis thaliana*. *TaKNOX11-A* overexpressing plants had decreased malondialdehyde content and increased proline content, allowing for more effective adaptation of plants to unfavorable environments.

**Conclusions:**

We identified TALE superfamily members in wheat and conducted a comprehensive bioinformatics analysis. The discovery of the potential role of *TaKNOX11-A* in drought resistance and salt tolerance provides a basis for follow-up studies of wheat TALE family members, and also provides new genetic resources for improving the stress resistance of wheat.

**Supplementary Information:**

The online version contains supplementary material available at 10.1186/s12864-022-08324-y.

## Background

Homeobox genes encode a large family of transcription factors (TFs), which are widespread in eukaryotes and essential in the growth and development of animals and plants. The first homeobox gene was found in fruit flies [1, 2]. In 1991,Vollbrecht et al. discovered the first homeobox gene KNOTTED-1 in maize [3]. A typical homeobox domain has a triple helix region composed of 60 amino acids. The first and second helix form a loop structure, and the second and third helix form a helix-turn-helix structure [4]. Homeobox genes in plants have been classified two different ways. Initially, homeobox genes were divided into seven classes, ZM-HOX, HAT1, HAT2, ATHB8, GL2, KNOTTED-like homeodomain (KNOX/KNAT), and BEL1-like homeodomain (BELL/BLH) [5]. In subsequent research, homeobox genes were subdivided into 11 classes, including HD-ZIP (I ~ IV), WOX, NDX, PHD, PLINC, LD, DDT, SAWADEE, PINTOX, KNOX/KNAT, and BLH/BELL [6].

According to protein sequences and evolution, KNOX and BEL1-like belong to the three-amino-loop-extension (TALE) superfamily. The TALE family functions directly in regulating plant growth and development [7–9], maintaining organ morphology [10], hormone regulation [11], signal transduction [12], tuber formation [13], and resisting abiotic stress [14]. KNOX protein usually has four domains: KNOX1, KNOX2, ELK, and homeodomain. In previous studies, the KNOX family was divided into two classes based on gene structural characteristics and expression patterns. KNOX proteins lacking homeodomains were found in dicots, and thus divided into three classes [7, 15]. The first class includes STM, KNAT1, KNAT2, and KNAT6; the second class includes KNAT3, 4, 5, and 7; and the third class contains KNATM, which are only found in dicotyledonous plants. The BELL family includes BEL1, ATH1, BLH1, BLH2, BLH3, BLH4, BLH5, BLH6, BLH7, BLH8, BLH9, and BLH10, which are not systematically classified [8].

In the TALE superfamily, research on KNOX genes is extensive. KNOX Class I genes are mainly expressed in meristems and have different expression patterns and functions [7, 16, 17]. For example, the *Arabidopsis KNAT2* gene is expressed in apical meristems, inducing homeotic conversion from nucellar to carpel-like structure and affecting the ectopic expression of AGAMOUS (AG) in the ovule center and carpel [18]. The *Arabidopsis STM* gene is involved in the formation of shoot apical meristem [17] while the *KNAT1* gene is involved in root tilt. *KNAT1* mutation significantly reduces auxin transport in basal leaves and increases the accumulation of auxin in the roots. The change in auxin transport is accompanied by a decrease in the level of PIN2 in the root tip. These results indicate that *KNAT1* may negatively regulate root tilt by regulating auxin transport [17].

Unlike the KNOXI subfamily, the KNOXII subfamily is expressed in a variety of tissues. Among the members of KNOXII, *KNAT7* has been studied the most. *KNAT7* regulates the negative feedback loop of secondary cell wall (SCW) biosynthesis, inhibiting the improper metabolic commitment of SCW formation, thereby maintaining metabolic homeostasis [19]. Recent studies have shown that *KNAT3* and *KNAT7* can form heterodimers, and KNAT3 can interact with the key SCW-forming TF NST1/2 to form a KNAT3-NST1/2 heterodimer complex, which regulates F5H and promotes lilac wood synthesis. This result indicated that *KNAT3* and *KNAT7* can jointly promote SCW biosynthesis [20]. Additionally, a study in *Medicago truncatula* found that *KNAT3/4/5-like* TFs may activate the EFD/RR4 pathway, locally limiting cytokinin signaling to prevent further rhizobia infection and nodule formation, thereby controlling nodular organ border and shape [21]. The third class of KNOX protein includes KNATM, whose members are only found in dicotyledonous plants. KNATM can interact with other TALE members to regulate their activities, and affect leaf polarity and development [15].

Compared with KNOX-like proteins, BELL-like proteins are less studied. In the BELL family, functions have been determined for ATH1, BEL1, SAW1/BLH2, SAW2/BLH4, PNF/BLH8, and PNY/LSN/BLR/VAN/RPL/RPL/BLH9. Functions of the other BELL-like members are unclear. The BELL protein ATH1 is a flower inhibitor, which can regulate the expression level of FLOWERING LOCUS C (FLC) [22]. In addition, *BLH3* and *BLH6* were shown to have opposite roles in the transition of flowers. Plants overexpressing *BLH6* had delayed flowering, while plants overexpressing *BLH3* flowered earlier than *BLH6* [23]. PNY inhibits flowering when interacting with ATH1, and promotes flowering when interacting with PNF [24]. BLH2/SAW1 and BLH4/SAW2 change leaf shape by inhibiting the expression of one or more KNOX genes to inhibit the growth of specific leaf subdomains [25]. BLH6 is a transcription repressor and the physical interaction of BLH6-KNAT7 enhances their inhibitory activity. BLH6-KNAT7 acts as a direct target through REV, binding to the REV promoter and inhibiting REV expression to control SCW biosynthesis in interfascicular fibers [26]. Large-scale yeast two-hybrid experiments with 13 BELL members in *Arabidopsis* showed that they all interact with at least one KNOX protein. For example, AtBLH1 protein and AtKNAT3 protein coordinately regulate *Arabidopsis* seed germination and seedling development [27]. Some GhBEL1-like proteins were found to interact with GhKNAT7 homologues and affect the cotton fiber SCW biosynthetic network [28].

Whole genome analysis of the TALE family has been performed on a variety of plants. These studies have identified *TALE* genes in *Arabidopsis* [8], poplar [29], cotton [28], pomegranate [30], and soybean [31]. However, *TALE* genes in wheat have not been systematically identified or investigated. As one of the most important crops in the world, wheat is an important source of human protein and mineral intake [32, 33]. With global climate change, it is essential to ensure the growth and development of wheat and to improve the resistance of wheat to various environmental and abiotic stresses. Recent studies have shown that the TALE family is not only important in regulating plant growth and development, but also functions in plant stress. For example, GmBHL4 protein heterodimerizes with GmSBH1 protein and regulates soybean response to drought, high temperature, and humidity stress [14]. A later study of the soybean TALE family showed that *TALE* genes potentially function in the response to abiotic stress and contribute to the genetic improvement of soybean resistance to salt and dehydration stress [31]. Among the 35 *TALE* genes in poplar, about 1/3 of the genes respond to salt stress [29]. Therefore, it is of great significance to study wheat *TALE* genes.

Here, we conducted a comprehensive genome-wide analysis of the TALE family in wheat and a total of 70 *TALE* genes were identified. Subsequently, we conducted a phylogenetic analysis and determined their physical location in the chromosome, homology, gene structure, and tissue-specific expression patterns. This study will help us to better understand the evolution of the *TALE* genes and their role in the growth, development, and regulation of responses to abiotic stress in wheat.

## Results

### Identification, characterization, and phylogenetic analysis of wheat *TALE* genes

**Fig. 1:**
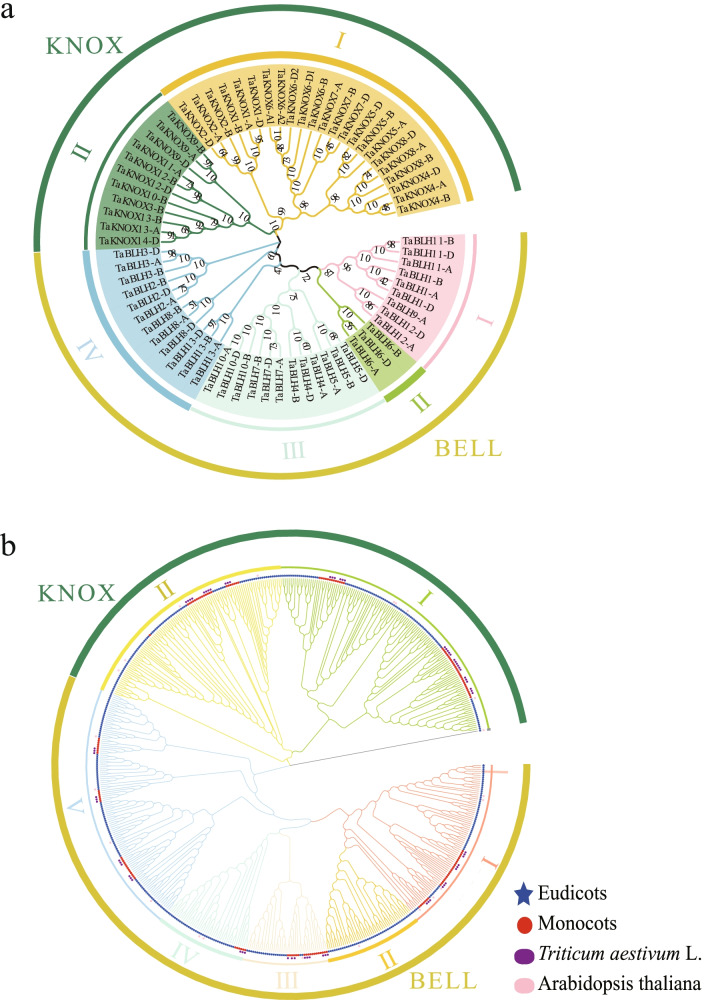
Phylogenetic analysis of TALE proteins. **a** Unrooted neighbor-joining tree constructed from wheat TALE proteins. **b** In total, 575 TALE protein sequences in 21 species (five monocotyledons, 16 dicotyledons) were used to construct the unrooted neighbor-joining tree. Different branches are marked with different colors. Eudicots and monocots are also distinguished by different color patterns (Eudicots: blue star, Monocots: red circle). Wheat and *Arabidopsis* proteins are labeled. (Wheat: purple, *Arabidopsis:* pink)

A total of 70 *TALE* candidate genes have been identified in wheat, of which 34 are *KNOX* genes and 36 are *BEL1-like* genes (Fig. 1a). *TALE* genes are named based on homologous gene pairs and locations on chromosomes. Although these genes all belong to the TALE family, their sizes and physicochemical properties vary greatly among different sub-families. Detailed information about these *TALE* genes is summarized in Additional file 9: Table S9.

The 70 *TALE* candidate genes are divided into two subfamilies. The length of the coding sequence (CDS) region of *KNOX* genes ranges from 462 bp (*TaKNOX7-A*) to 1170 bp (*TaKNOX13-A*), and the average length is 938 bp. The molecular weight of *KNOX* genes ranged from 16.54 kDa (*TaKNOX7-A*) to 42.48 kDa (*TaKNOX13-A*) with an average weight of 34.33 kDa. The isoelectric point (pI) values of these genes ranges from 5.09 (*TaKNOX13-B*) to 9.11 (*TaKNOX6-B*), with 88% of members (30/34) exhibiting acidic pI values. The length of the CDS region of *BEL1-like* genes ranges from 1119 bp (*TaBLH8-A*) to 2412 bp (*TaBLH6-D*), and the average length is 1782 bp. The molecular weight of *BEL1-like* genes ranged from 40.06 kDa (*TaBLH8-A*) to 84.41 kDa (*TaBLH6-D*), with an average weight of 63.93 kDa. The pI values of these genes ranges from 5.23 (*TaBLH7-A*) to 8.34 (*TaBLH2-A*, *TaBLH2-B*, *TaBLH2-D*) with 78% members (28/36) exhibiting acidic pI values. KNOX subfamily members thus have a smaller molecular mass compared with BEL1-like subfamily members. Most of the members of the two subfamilies are acidic, and are located on the nucleus based on predictions of subcellular location.

In order to study the phylogeny and taxonomic relationships of *TALE* family genes, a phylogenetic tree was constructed with 575 conserved domains of TALE proteins in 21 species (five monocotyledons, 16 dicotyledons) (Fig. 1b). We also constructed an unrooted phylogenetic tree containing only wheat TALE proteins. As expected based on the similarity of protein sequences and previous studies in *Arabidopsis*, the TALE family is divided into two major branches, KNOX proteins and BEL1-like proteins [7, 15, 34]. KNOX proteins can be divided into three subclasses, class I (KNAT1, 2, 6, STM), class II (KNAT3, 4, 5, 7), and class III (KNATM), which is unique to dicotyledons. BEL1-like proteins are divided into five subclasses, class I (BLH3/5/6/7/10), class II (BLH1), class III (BEL1), class IV (BLH2/4), and class V (BLH8/9/11/ATH1). Most TALE proteins of dicotyledons and monocotyledons gather in the same branch. TALE proteins of the same species also cluster in the same branch. Wheat TALE proteins are divided into six classes (KNOXI, KNOXII, BLH3/5/6/7/10, BEL1, BLH2/4, BLH8/9/11/ATH1) without BLH1 or KNATM proteins. Class KNOXI contains the most family members accounting for about 33%, and the BLH2/4 class contains the fewest family members accounting for about 4%. In addition, wheat has more KNOX1 proteins compared to other species, indicating that wheat TALE family may have expanded in the KNOXI class.

### Chromosomal location

**Fig. 2:**
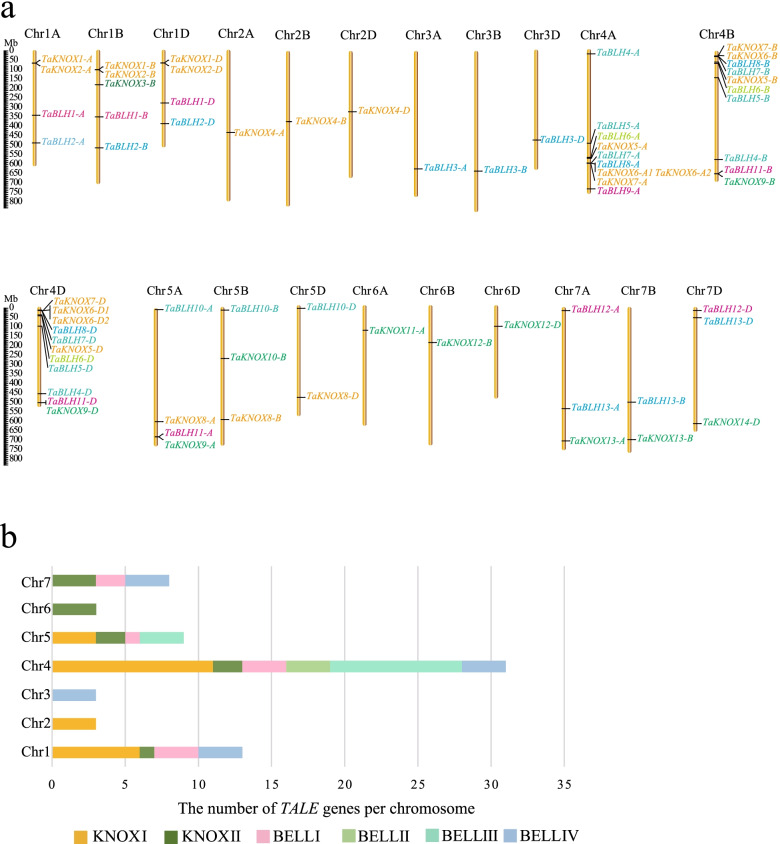
Chromosomal distribution of wheat *TALE* genes. **a** Distribution map of 70 wheat *TALE* genes on wheat chromosomes with the name of the gene on the right side. The scale is in mega bases (Mb). Different subclass genes are distinguished by different colors. **b** The number of *TALE* genes on different chromosomes (Chr1–Chr7)

All 70 *TALE* genes are located on 21 chromosomes (Fig. 2a and Additional file 9: Table S9). These members are unevenly distributed across the genome. Chromosomes 1 to 7 respectively contain 13, 3, 3, 31, 9, 3, and 8 genes. There is a cluster of genes on chromosome 4, with the largest distribution of members (about 44%), covering all KNOX and BEL1-like classes (Fig. 2b). These results indicate that the replication event of the wheat TALE family may have occurred during the formation of chromosome 4, as members of the same subclade tend to gather in the same chromosome, and the evolution of each chromosome is relatively independent.

### Duplication and syntenic analyses in wheat *TALE* genes

**Fig. 3:**
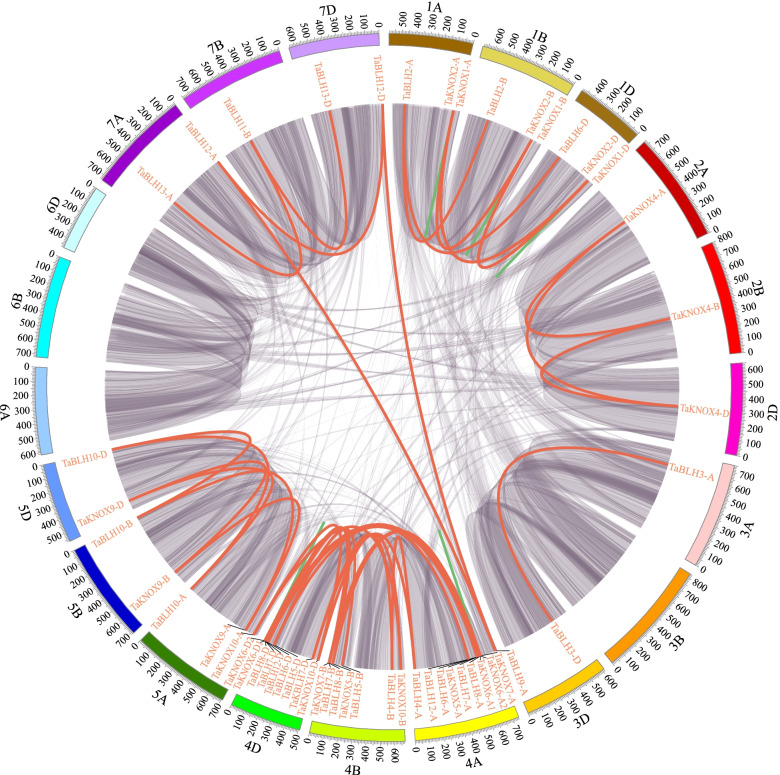
Collinearity analysis of the *TALE* gene family in the wheat genome. The analysis results are displayed in the Circos plot. Different chromosomes are represented by different colors, and the length of the chromosomes is marked. The segment and tandem repeats of the *TALE* genes are mapped in the wheat genome. Red lines represent duplicate *TALE* gene pairs. Green lines represent the tandem *TALE* gene pair and the purple area represents the synonymous blocks in the wheat genome

In order to explore the reasons for the expansion of wheat *TALE* genes, we analyzed homology groups and duplication events in the wheat genome (Table 1 and Additional file 10: Table S10). Most of the wheat *TALE* genes show a 1:1:1 homology, where three *TALE* genes localized on the A, B, and D sub-genomes and shared high homology, which we refer to as triplets. The proportion of homeologous triplets in the wheat TALE family is close to twice the proportion of homeologous triplets in the wheat genome (35.8%). However, the proportions of homologous specific duplications (0%), loss of one homologous gene (8.6%), orphans or singletons (7.1%) are all lower than the proportions in the whole wheat genome (5.7, 13.2, 37.1%). This high proportion of homeologous triplets indicates that wheat polyploidization was the main cause of wheat TALE family expansion. Then we explored the tandem and segmental duplication events of *TALE* genes in the wheat genome (Fig. 3 and Additional file 11: Table S11). A total of 52 *TALE* genes are located in the wheat collinearity blocks, forming 58 pairs of duplicated genes. In total, 53 pairs were caused by segmental duplication events and five pairs were caused by tandem duplication events (*TaKNOX1-A*/*TaKNOX2-A*, *TaKNOX1-B*/*TaKNOX2-B*, *TaKNOX1D*/*TaKNOX2-D*, *TaKNOX6-A1*/*TaKNOX6-A2*, *TaKNOX6-D1*/*TaKNOX6-D2*). *TALE* genes have more segmental events in chromosome 4, which also explains why there are more family members on chromosome 4. In summary, the expansion of the *TALE* genes in wheat is mainly due to polyploidization and segmental duplication events.

**Table 1:** Homoeologous *TALE* genes in wheat

Homoeologous group (A:B:D)	All wheat genes	Class		Number of groups	Number of genes	% of genes
		KNOX	BELL			
1:1:1	35.8%	7	11	18	54	77.2%
1:1:0/1:0:1/0:1:1	13.2%	2	1	3	6	8.6%
1:1:n/1:n:1/n:1:1, *n* > 1	5.7%	–	–	–	–	0%
Orphans/singletons	37.1%	4	1	5	5	7.1%
Other rations	8.0%	1	–	1	5	7.1%
Total	99.8%	14	13	27	70	100%

**Fig. 4:**
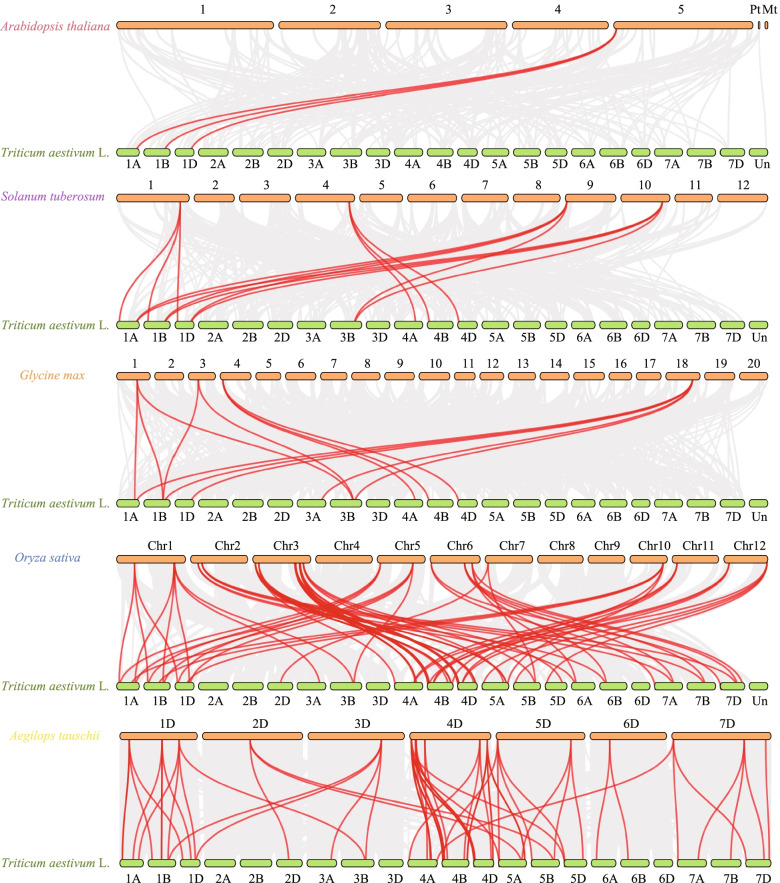
Syntenic relationships of wheat *TALE* genes among *Arabidopsis thaliana*, *Glycine max*, *Zea mays*, *Oryza sativa*, and *Aegilops tauschii*. Genomic collinearity regions of wheat and other species are indicated by gray lines. The red lines indicate the syntenic *TALE* gene pairs

In order to further explore the evolutionary clues of *TALE* genes in wheat and other species, six plants (three dicotyledons and three monocotyledons) were analyzed for genome collinearity. The results of collinearity analysis are shown in Fig. 4. We also counted the non-redundant collinear genes between wheat and five other species. We found 3, 13, 13, 68, and 62 collinear gene pairs in wheat with *Arabidopsis thaliana*, *Solanum tuberosum*, *Glycine max*, *Oryza sativa*, and *Aegilops tauschii*, respectively. Fig. S1a shows the UpSet plot of non-redundant genes in different species. Three wheat TALE members have collinear pairs in the three dicot species, and 48 wheat TALE members have collinear pairs in two monocot species. Three wheat TALE members (*TaBLH2-A*, *TaBLH2-B*, *TaBLH2-D*) have collinear pairs in all five species. Wheat thus has more collinear pairs with rice and *Aegilops tauschii*, which are also monocots. These results are helpful for studying the evolution of wheat *TALE* genes. We speculate that the wheat *TALE* genes may be derived from homologous genes of other species.

Then we calculated the Ka/Ks value of the wheat *TALE* orthologous gene pairs among *Triticum aestivum* and the two species to study the selection pressure of the TALE family during evolution. The Ka/Ks values were all less than 1, with an average of 0.22 (Fig. S1b). This result indicated that the *TALE* family genes are continuously evolving through purification and selection.

### Codon usage pattern analyses in wheat *TALE* genes

**Fig. 5:**
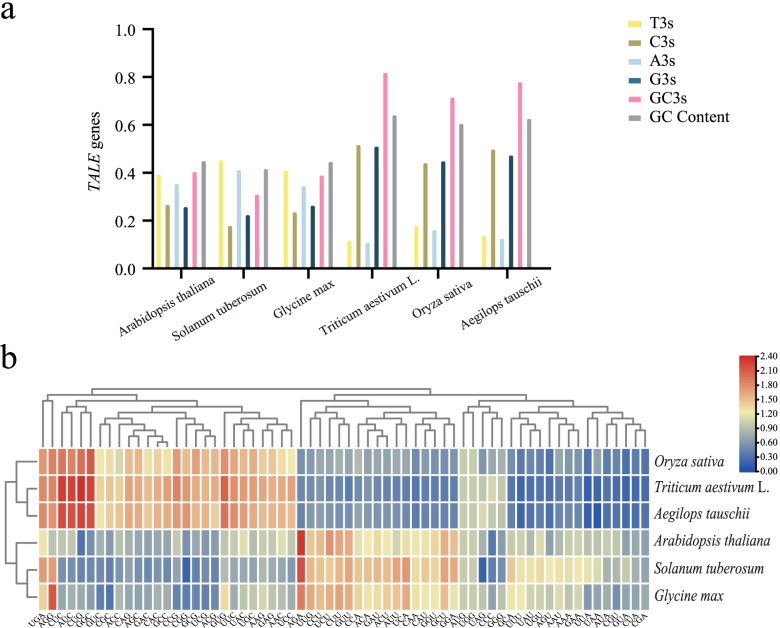
Codon usage pattern analyses in wheat *TALE* genes. **a** A bar graph representing the content of different bases in the third position of codons in six species. **b** The heatmap shows the relative synonymous codon usage (RSCU) values of the CDS sequence of cystatin in six species

Codons are very important in the transmission of biological information. Synonymous codons refer to multiple codons that encode the same amino acid [35, 36]. The frequency of synonymous codon usage varies greatly in different species [37]. Synonymous codon preference can be used as an important parameter of species evolution. Codon Usage Bais (CUB) can affect the translation efficiency of genes and thus affect gene expression [38, 39]. The codon usage pattern is believed to be related to the GC content of the third codon position (GC3) [40]. We used the CDS sequences of *TALE* genes from six species to analyze the codon usage patterns of *TALE* genes in different species (Table 2). We observed that the average GC ratio of *TALE* genes in monocots is higher than that in dicots. The results also showed that the average ratio of A/T-terminated codons of the monocotyledon was low, while the average ratio of G/C-terminated codons was relatively high (Fig. 5a). These results are also in line with previous research [41, 42]. Among several species, wheat has the lowest average effective number of codon (ENC) and the highest GC content. This shows that the cub of wheat is the strongest. Relative Synonymous Codon Usage (RSCU) can indicate cub more intuitively [43]. RSCU > 1 means higher codon usage, RSCU = 1 means no preference for codon usage, and RSCU < 1 means low frequency of codon usage [44]. Subsequently, we conducted a relative (RSCU) analysis of the *TALE* genes in the six plants, and used the RSCU value to draw a heat map (Fig. 5b). We found that RSCU values are similar in monocots and dicots. *Triticum aestivum*, *Oryza sativa,* and *Aegilops tauschii* are gathered into one category. *Arabidopsis*, *Solanum tuberosum,* and *Glycine max* are clustered into one category. This may be related to the evolutionary relationship of species.

**Table 2:** Codon usage indicators of the TALE family in six different species

Species Name	CBI	Fop	ENC	GC3S	GC Content
*Arabidopsis thaliana*	−0.017	0.423	54.55	0.407	0.452
*Solanum tuberosum*	− 0.121	0.362	50.48	0.312	0.419
*Glycine max*	−0.078	0.386	53.02	0.392	0.449
*Triticum aestivum L.*	0.198	0.542	41.66	0.813	0.644
*Oryza sativa*	0.154	0.518	48.27	0.718	0.608
*Aegilops tauschii*	0.191	0.538	44.01	0.782	0.629

Abbreviations: *CBI* codon bias index, *Fop* frequency of optimal codons, *ENC* effective number of codon; and *GC3s* contents of G or C bases at the third position of the codons, *GC content* the contents of the G and C bases of the codons

### Conserved protein motifs and structure of wheat *TALE* genes

**Fig. 6:**
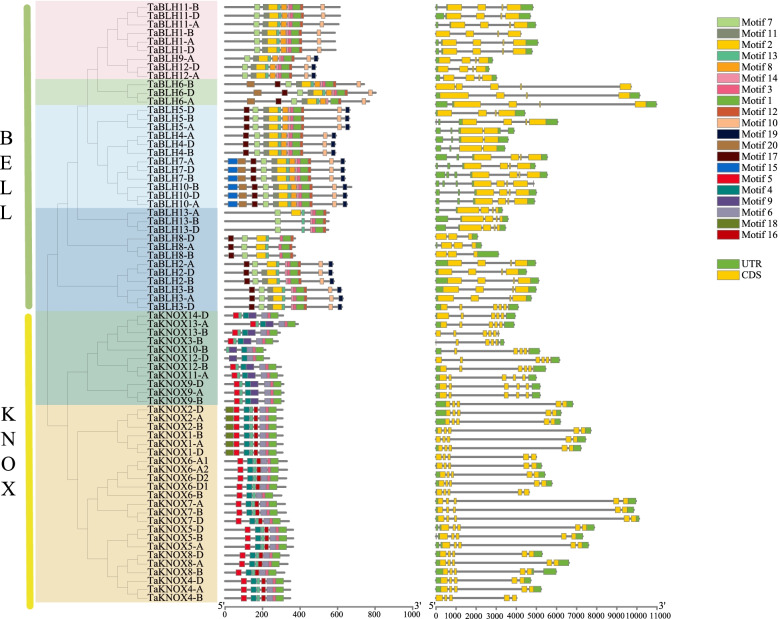
Evolutionary tree, conserved motif compositions, and gene structures of 70 wheat *TALE* genes. The unrooted neighbor-joining tree was constructed by MEGA7. The 20 motifs were identified by MEME Suite. Different conserved motifs are displayed in different colored boxes. Motif 5 represents KNOX domain, motif 4 represents KNOX domain, motif 6 represents ELK domain, motif 7 represents POX domain, and motif 1 represents HOX domain. The lengths of the exons and introns of each gene are shown proportionally. The yellow boxes represent exons, the black lines represent introns, and the green boxes represent 5′ and 3′ non-coding regions

In order to gain a deeper understanding of the diversity of *TALE* gene functions, we used MEME online software (MEME, http://meme-suite.org/tools/meme) to predict conserved motifs in wheat TALE proteins. A total of 20 conserved motifs were identified. The KNOX and BEL1-like subfamilies respectively contain eight and 14 motifs. Motif 1 (HOX) is shared by the two subfamilies. As anticipated, some motifs are specific to each family. For example, motif 5 (KNOXI conserved domain), motif 4 (KNOXII conserved domain), motif 6 (ELK conserved domain), and motifs 9, 16, and 18 exist in the KNOX subfamily, and motif 7 (POX conserved domain) as well as motifs 2, 8, 10, 11, 12, 14, 15, 17, 19, and 20 exist in the BEL1-like subfamily (Fig. 6 and Additional file 12: Table S12). Differences in protein motifs among subfamilies may explain the functional diversity of KNOX and BEL1-like family proteins. In general, in phylogenetic analysis, proteins with similar motifs tend to cluster together, which means that members of the same subclade have similar functions.

In order to investigate gene structure, we used the GSDS online tool (http://gsds.cbi.pku.edu.cn/) to analyze the intron-exon structure of wheat *TALE* genes (Fig. 6). We found that the number of introns in wheat *TALE* genes ranges from 2 to 7, and the number of exons ranges from 3 to 8. The numbers of introns and exons in the KNOX subfamily are slightly higher than the numbers of introns and exons in the BEL1-like family, and the lengths of exons in the KNOX family are significantly longer.

### Cis-elements analysis and expression profiles analysis of wheat *TALE* genes

**Fig. 7:**
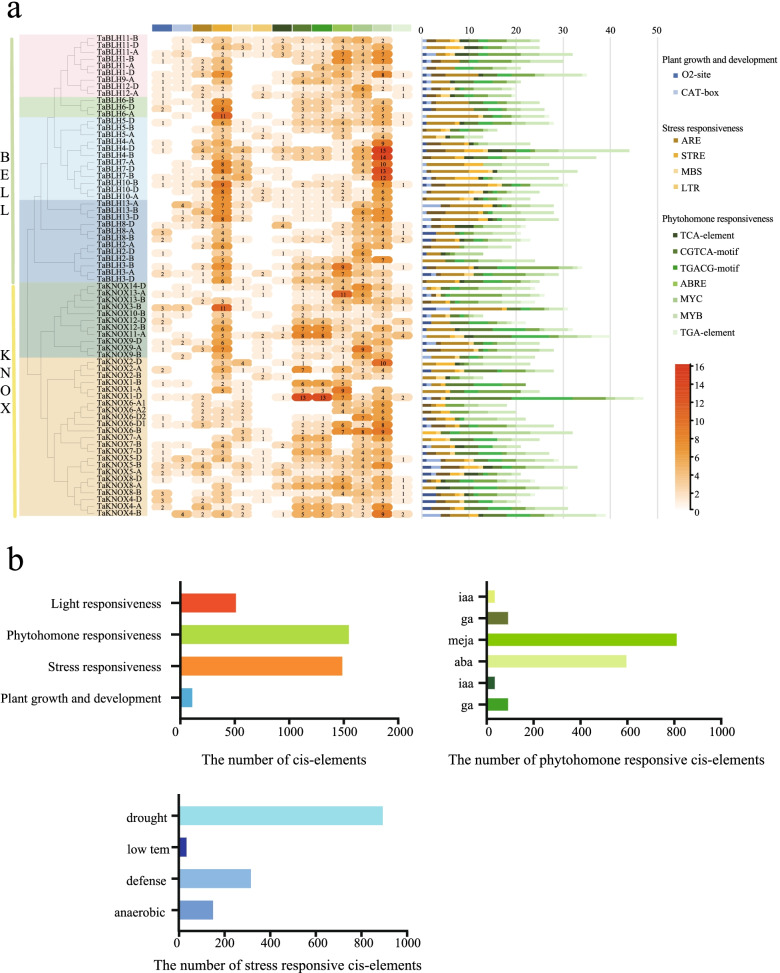
Cis-acting elements of wheat *TALE* genes. **a** Number of cis-acting elements per *TALE* genes heatmap; different colors indicate the number of cis-acting elements; white means no cis-acting elements. The column icon on the right indicates the number of cis-acting elements for each *TALE* gene. Different types of cis-acting elements are shown in different colors. **b** The number of different cis-elements in wheat *TALE* genes

In order to further understand the potential regulatory mechanism of *TALE* genes, and which plant hormones, defense, and stress response elements regulate these genes, we used the PlantCARE web server (http://bioinformatics.psb.ugent.be/webtools/) to search for possible cis-elements in the 2000 bp promoter region of wheat *TALE* genes (Fig. 7a and Additional file 13: Table S13). Most of the identified cis-acting elements were hormone response factors (42.1%), followed by environmental stress response elements (40.4%), photosensitive elements (14.2%), and plant growth-related elements (3.3%). Hormone response elements were mostly methyl jasmonate (MeJA) response elements (52.3%), followed by abscisic acid (ABA) response elements (38.6%), while gibberellin (GA) and 3-indoleacetic acid (IAA) response elements were lowest. Among the elements of environmental stress response, most are related to drought response (60%), followed by defense response (21.5%) (Fig. 7b). These results indicate that wheat *TALE* genes are likely to be related to the response to abiotic stress, especially drought.

**Fig. 8:**
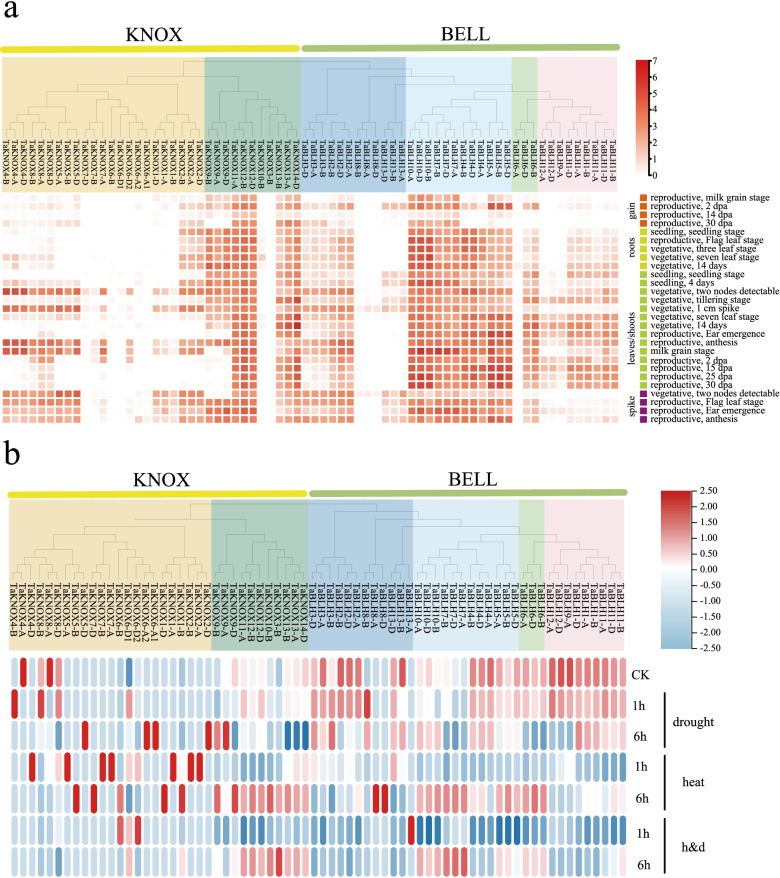
Heat map of wheat *TALE* genes expression in different developmental stages and abiotic stresses. **a** The expression levels of wheat *TALE* genes were downloaded from the wheat expression database. Log2 (tpm + 1) was used to build the heatmap. **b** Heat map of *TALE* genes expression in wheat under control, drought, heat, and drought + heat treatments

In order to understand the expression profiles of *TALE* genes in different tissues and growth periods of wheat, we downloaded the RNA-seq data of roots, stems, leaves, and ears in the vegetative growth and reproductive growth stages of Chinese spring wheat seedlings from the wheat expression browser (http://www.wheat-expression.com) (Additional file 14: Table S14) and generated a tissue-specific expression heat map (Fig. 8a). We found that 14.3% (10/70) of *TALE* genes showed very low expression or no expression (log2(tpm + 1) < 1) at all developmental stages, and the KNOXI branch contained the most genes with low expression. The expression of KNOXI genes was relatively low in the developmental stages of seeds and roots, which is consistent with the results of previous studies showing that KNOXI members are expressed in meristems, which is necessary for the development and maintenance of meristems [45].

BELLII, BELLIII, and KNOXII also contain some genes with low expression, indicating that the genes of this subfamily may have experienced strong functional differentiation and redundancy. Other genes are expressed in at least one developmental stage of seeds, roots, leaves/shoots, and spikes. Among them, BELLIII family genes and most genes in the KNOXII family have high transcription levels at all stages. *TaKNOX11-A*, *TaKNOX12-A*, and *TaKNOX12-B* have the highest expression in KNOXII. Previous studies have shown that *KNOXII* genes are involved in regulating the secondary growth of plant cell walls and are essential in the development of roots, stems, seed coats, and carpel. Therefore, these genes are likely to participate in the formation of plant SCWs and to function in improving plant stress resistance.

In order to study the expression of *TALE* genes under abiotic stress, we downloaded the relative expression abundance of all *TALE* genes in the leaves of 7-day-old seedlings under drought and heat stress from the wheat expression browser. We analyzed the expression levels of *TALE* genes under different physiological conditions and drew a heat map of the expression levels (Fig. 8b). From the overall trend, under drought and heat stress, most of the BELL family members were down-regulated, and most of the KNOX family members were up-regulated compared with the control. The similar structures among members may lead to their functional similarity. These results indicate that abiotic stress can significantly induce multiple *TALE* genes.

**Fig. 9:**
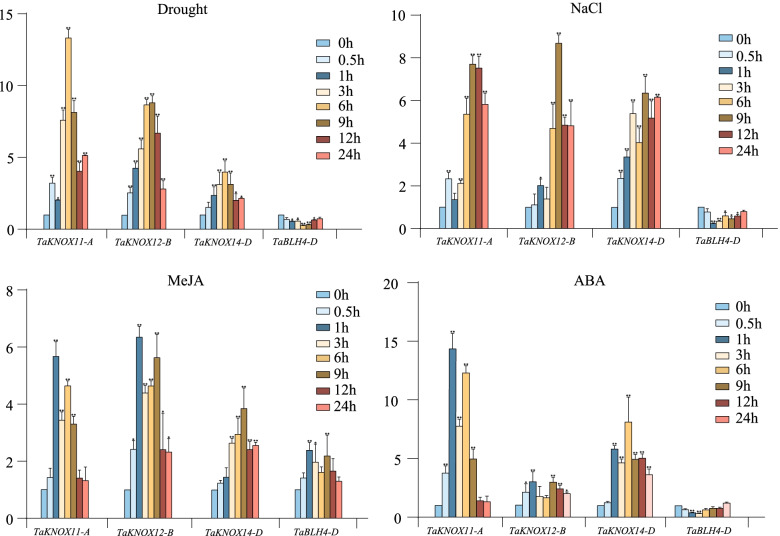
Expression levels of four wheat *TALE* genes under (**a**) drought, (**b**) NaCl, and (**c**) MeJA treatment. Three biological replicates were done for each treatment. The error bars indicate the SD of the three replicates. Asterisks indicate significant differences between WT, OE, and mutant lines (*p < 0.05, **p < 0.01, Student’s *t*-test)

We used qRT-PCR to detect the expression levels of four *TALE* genes under drought, salt, MeJA, and ABA stress (Fig. 9). Because these four genes all contain more cis-acting elements, and their expression changes greatly under stress treatments. The expression levels of *TaKNOX11-A*, *TaKNOX12-B* and *TaKNOX14-D* were all up-regulated under the four abiotic stress treatments. Among them, the overall expression level of *TaKNOX11-A* under the four stresses was higher than that of the other genes. The expression of *TaKNOX11-A* reached the highest value after 6 h of drought treatment, 9 h of salt stress, 1 h of MeJA stress, and 1 h of ABA stress. All four genes showed an up-regulation trend under MeJA treatment. *TaBLH4-D* showed a down-regulation trend under all three stresses except MeJA. We selected *TaKNOX11-A* for subsequent analysis because its expression levels were significantly up-regulated under the four stresses.

### Protein–protein network analysis and GO annotation of wheat *TALE* genes

Interaction network analysis helps to understand the biological functions and molecular mechanisms of proteins. Therefore, we used STRING to predict the proteins that interact with wheat TALE proteins (Fig. S2). According to the prediction results, 32 TALE family members have interactions, and four proteins encoded by other genes that do not belong to the TALE family also interact with TALE proteins. In terms of the interactions of TALE family members, KNOX interacted with members of the BEL1-like subfamily. This is also consistent with the conclusion of previous studies where BELL and KNOX proteins specifically recognize and bind to form a KNOX-BELL heterodimer protein [46]. *TaKNOX11-A* can interact with six other TALE family members (*TaBLH2-A*, *TaBLH6-A*, *TaBLH6-B*, *TaBLH6-D*, *TaBLH11-B*, *TaBLH12-A*, and *TaBLH12-D*), most of which belong to the BLH1 class of proteins. In previous studies, KNAT3 proteins were shown to interact with BLH1 proteins, affecting the response of plants to ABA, which may also indirectly affect the resistance of plants to adversity [27]. TaKNOX9-B and TaKNOX9-D can interact with members of four OFP2 protein families. In rice, OFP2 and KNAT7 work together in a manner similar to OFP2-KNAT7-BLH6 to inhibit SCW biosynthesis [47]. These results provide valuable information for further identification of the function of *TALE* genes.

We performed gene ontology (GO) annotation to further study the biological processes related to *TALE* genes in wheat (Fig. S3 and Additional file 15: Table S15). *TALE* genes are involved in a variety of biological processes, such as biosynthetic processes (GO:2001141), regulation of metabolic processes (GO:0051252), regulation of gene expression (GO:0010468), response to hormones (GO: 0009725), and cell response to stimulation (GO:0051716). For molecular functions, *TALE* genes are involved in DNA binding (GO:0003677), nucleic acid binding (GO:0003676), heterocyclic compound binding (GO:1901363), and organic cyclic compound binding (GO:0097159). Analysis of cell components showed that in addition to the nucleus, TALE proteins may also be located in intracellular organelles, membrane boundary organelles, and cytosol. The above results indicate that as TFs, wheat TALE family members participate in different developmental and metabolic processes, and may respond to adversity, providing further support for the analysis results of cis-acting elements.

### Overexpression of *TaKNOX11-a* affects seed germination and root length of *Arabidopsis thaliana* under drought and NaCl stress

To study the effects of *TaKNOX11-A* on plants under drought or salt stress, we selected three homozygous T3 generation *TaKNOX11-A* overexpressing *Arabidopsis* lines (OE1, OE2, and OE3) with the highest expression levels and *knat3* mutant plants for follow-up experiments. We cultured sterilized seeds on MS medium with no treatment, 8% PEG6000, 10% PEG6000, 100 mM NaCl, and 150 mM NaCl. The germination rate of seeds on MS medium with no treatment was used as a control (Fig. S4a and Additional file 16: Table S16). There was no significant difference in the germination rates of WT, OE1, OE2, OE3, and *knat3* seeds on MS medium (Fig. S4b). In MS medium containing 8% PEG6000 and 10% PEG6000, the germination rates of several strains were different, and the germination rate of the overexpression strain was higher than that of WT and *knat3*. Compared with the MS medium, the germination rate of several strains was inhibited by 8 and 10% PEG6000 treatment (Fig. S4b). In the MS medium containing 100 mM NaCl and 150 mM NaCl, the germination rates of several strains were more severely inhibited (Fig. S5a and Additional file 16: Table S16), but the germination rate of the overexpression strain was still the highest, followed by the germination rates of WT and *knat3*. The germination rate of *knat3* was the lowest overall (Fig. S5b).

### *TaKNOX11-a* enhances the drought and salt tolerance of *Arabidopsis*

In subsequent root length and fresh weight experiments, we found that under drought and salt stress, the root lengths of several lines were shorter than the control because they were inhibited (Fig. S6a, Fig. S6b and Additional file 17: Table S17). Root fresh weight also decreased (Fig. S6c). Under drought and salt stress, the root length and fresh weight of the three overexpression lines were highest, while the root length and fresh weight of *knat3* were the lowest among several strains. In general, under drought and salt stress, the three overexpression lines had heavier fresh weight and longer roots, followed by WT, while *knat3* had the shortest roots and lowest fresh weight. Therefore, the *TaKNOX11-A* gene may affect the ability of plants to resist drought and salt stress.

To explore the effects of *TaKNOX11-A* on plants under drought and salt stress treatments, we performed drought and salt stress treatments on two-week-old WT, OE1, OE2, OE3, and *knat3* plants (Fig. S7a, Fig. S8a). We counted the survival rates of the four lines and measured the amounts of proline (PRO) and malondialdehyde (MDA) (Additional file 17: Table S17). After drought and salt stress treatments, seedlings of wild-type and *knat3* plants appeared to appeared to be in poorer physical condition compared to the *TaKNOX11-A*-overexpressing lines. It can be seen from Fig. S7a and Fig. S8a that wild-type and *knat3* plants are badly wilted, while the three *TaKNOX11-A* overexpression lines have less wilted. After re-watering, the *TaKNOX11-A* overexpression lines had more plants surviving. The experimental results showed that under the two stress treatments, the survival rate of the three overexpression lines was higher than that of WT and *knat3* (Fig. S7b, Fig. S8b). The *knat3* mutant plant had the lowest resistance to stress, followed by WT. Among several lines, the PRO content of the overexpression line was the highest, and the PRO content of the *knat3* line was the lowest (Fig. S7c, Fig. S8c). The MDA content results of the five strains were opposite to the PRO content results, and the MDA content of the three overexpression lines was lower than that of WT and *knat3* (Fig. S7d, Fig. S8d).

## Discussion

*TALE* superfamily genes are widely present in plant genomes and are essential for regulating plant development, growth, and stress response. At present, whole genome analysis of *TALE* genes has been carried out in a variety of plants, such as *Arabidopsis* [8], poplar [29], cotton [28], pomegranate [30], and soybean [31]. However, there has not been a systematic analysis of this gene family in wheat. This study explored the phylogeny and evolution of wheat *TALE* genes through a comprehensive and systematic analysis, and investigated the expression of *TaKNOX11-A* in *Arabidopsis* under drought and salt stress. A total of 70 *TALE* genes were identified in wheat. The number of members of the TALE superfamily varies from species to species. The number of *TALE* genes in the wheat genome is about three times that of *Arabidopsis* (22) and rice (22), and twice that of poplar (35). The proportion of homologous triplets in the wheat TALE family is close to twice the proportion of homologous triplets in the wheat genome (35.8%). This high retention rate can partly explain why the number of *TALE* genes in wheat is higher than that of other species.

In order to explore the evolutionary relationships among TALE proteins, we constructed a phylogenetic tree of 21 species (five monocotyledons, 16 dicotyledons) (Fig. 1). Referring to the classification of the TALE superfamily in *Arabidopsis*, we divided the wheat TALE members into the KNOX subfamily and the BEL1-like subfamily. The KNOX subfamily is further divided into three classes, and the KNATM class is unique to dicots. We speculated that *KNATM* genes may have been lost during the evolution of monocots. The BEL1-like family has not been systematically classified in previous studies. Here we divided it into five classes based on the clustering in the evolutionary tree. TALE members in the same branch from different species may have similar biological functions.

In the study of gene structure and conserved motifs, we found that wheat TALE members in the same branch of the evolutionary tree have similar gene structures and conserved motifs, supporting our classification results. Studies on the physicochemical properties of wheat show that KNOX members and BELL members are quite different. The amino acid length and molecular weight of BELL proteins are much larger than those of KNOX proteins. This is consistent with the conclusions in poplar, soybean, and cotton. For gene structure, the BEL1-like family has more introns. Consistent with the BEL1-like family in soybean, the introns of wheat BEL1-like family genes mostly appear in the 5′ UTR region. Introns are critical to the evolution and production of genes in new gene families [48, 49]. The different distributions of introns among members of the TALE family may affect the evolution of new family members.

In addition to introns that may affect the generation of new family genes, replication events between genomes also play a role. In the wheat genome, there are 58 pairs of replicated genes, 53 of which were produced by segmental replication. Combined with the high retention rate of homologous triplets in the wheat TALE family, polyploidization and fragment duplication are the main reasons for the expansion of wheat TALE family. The Ka/Ks ratio indicates that over evolutionary time, *TALE* genes have mainly undergone purification selection (Ka/Ks ratio < 1). This result indicates that the expansion of the wheat TALE family may increase its ability to adapt to the environment and help widen its distribution [50]. In some cases, gene duplication may lead to new functionalization and sub-functionalization [51].

Cis-elements exist in gene promoters and specifically bind with TFs to regulate gene transcription, and function directly in the regulation of plant gene expression. We found that the main cis-acting elements of wheat *TALE* genes are hormone response factors (42.1%), followed by environmental stress response elements (40.4%). Among hormone response elements, most were MeJA response elements (52.3%), followed by ABA response elements (38.6%). This result shows that the promoter of *TALE* genes has a certain degree of conservation. Among the elements of environmental stress response, most are related to drought response (60%), followed by defense response (21.5%). The GO annotation results also indicate that members of the wheat TALE family may respond to adversity, which provides further support for the analysis results of cis-acting elements. These results indicate that the wheat TALE family may participate in the response to drought stress through the ABA or MeJA pathway.

Subsequently, we selected four wheat *TALE* genes with higher expression levels in each growth period of wheat and more cis-acting elements for follow-up studies. We studied the gene expression levels of the four TALE members under hormone treatment and stress treatment. RT-qPCR analysis showed that drought, heat, NaCl, and MeJA treatments significantly induced *TaKNOX11-A* gene expression. We cloned the wheat *TaKNOX11-A* gene and overexpressed it in *Arabidopsis* to study its role in mediating the plant’s response to abiotic stress. We found that, compared with WT and mutant plants, transgenic *TaKNOX11-A Arabidopsis* plants cultured under drought or salt stress showed higher germination and survival rates, and longer roots. These findings indicate that *TaKNOX11-A* may be a positive regulator of drought resistance and salt tolerance in plants.

PRO can maintain dynamic plant balance under adverse conditions [52], increasing stress tolerance in plants [53]. For plants under drought or salt stress, the MDA level can also be used as an indicator of the degree of cell damage [54, 55]. We found that compared with WT, the accumulation of PRO was higher in *TaKNOX11-A* transgenic plants, and the accumulation of MDA was lower. This indicated that PRO may directly promote the enhanced tolerance of *TaKNOX11-A* transgenic plants to drought and salt stress. These physiological changes indicated that *TaKNOX11-A* has a positive role in the response of plants to adverse environmental conditions. However, it is not yet clear how *TaKNOX11-A* affects the function of other genes to increase drought tolerance in plants. Although exogenous MeJA and ABA significantly induce the expression of *TaKNOX11-A*, however, whether the function of *TaKNOX11-A* is mediated through the MeJA and ABA pathways is still unclear. The protein interaction network diagram shows that *TaKNOX11-A* can interact with many BLH1 proteins. Studies on *Arabidopsis* have shown that the expression of BLH1 and KNAT3 is enhanced under stress conditions. The increase of BLH1 promotes the retention of KNAT3 in the nucleus by masking the nuclear export signal (NES). The resulting BLH1/KNAT3 complex strongly binds to the ABI3 promoter region and enhances ABI3 production. The ABI3 TF has an important regulatory role in abiotic stresses such as temperature stress and salt stress [56–58]. Therefore, we need to further study and clarify the mechanism of *TaKNOX11-A* regulating abiotic stress response .

## Conclusion

This study identified 70 TALE family members from the wheat genome. We conducted a comprehensive and systematic bioinformatics analysis of these 70 *TALE* genes. The evolutionary mechanisms, gene structures, and expression patterns of wheat *TALE* genes were studied. We selected *TaKNOX11-A* for follow-up functional verification. Our results show that *TaKNOX11-A* improved tolerance of *Arabidopsis* plants to drought and salt stress. This study provided a theoretical basis for further research on the wheat *TALE* family and provided new genetic resources for wheat resistance studies.

## Methods

### Identifcation and characteristics of the *TALE* genes in wheat

Hidden Markov Models (HMMs) were employed to identify *TALE* genes in the wheat (*Triticum aestivum* L.) genome. A local protein database was constructed from the protein sequences of wheat under the Ensembl Plants database (http://plants.ensembl.org/index.html) [59]. In order to obtain the wheat *TALE* genes, we used a TALE (PF00046) to search the local protein database in the HMMER3 software (E value <E^− 10^). The identified candidate *TALE* genes were submitted to the SMART database (https://smart.embl-heidelberg.de/) [60] and the NCBI protein Batch CD-search database (http://www.ncbi.nlm.nih.gov/Structure/bwrpsb/bwrpsb.cgi) [61] in order to eliminate genes lacking the conserved domain. Then we selected genes with POX and HOX conserved domains as *BELL* genes, and genes with KNOX1 or KNOX2, and HOX conserved domains as *KNOX* genes. Ultimately, we acquired 70 *TALE* genes in wheat. Subsequently, the physicochemical parameters of TALE proteins were predicted in ExPASY (https://www.expasy.org/) [62].

### Phylogenetic analysis and classification of wheat TALE proteins

We extracted the conserved protein structures for multi-species phylogenetic analysis. Proteins for multiple species were downloaded from NCBI (https://www.ncbi.nlm.nih.gov/), the Ensembl Plants database, and TAIR (https://www.arabidopsis.org/). The method for identifying TALE proteins from multiple species was the same as the above method for identifying wheat TALE proteins. Phylogenetic trees of full-length amino acid sequences were produced using the neighbor-joining (NJ) method with 1000 bootstrap replications, Poisson model, and pairwise deletions in MEGA7 software (https://www.megasoftware.net/) [63].

Analysis of chromosomal location*TALE* genes were mapped to the 21 wheat chromosomes using the information obtained from the Ensembl Plants database using Mapchart software [64].

### Duplication and syntenic analyses between wheat *TALE* genes and those of several other species

Homoeologous genes were identified using the phylogeny [65] and the Ensembl Plants database. Segmental and tandem duplications were determined for the wheat *TALE* genes using McscanX software [66] and a pair of duplicated TALE proteins were defined based on the following three criteria: (1) the alignment covered > 80% of the longer gene; (2) the aligned region had an identity > 80%; (3) only one duplication event was counted for tightly linked genes [67, 68]. Tandem and segmental duplication events were divided according to the chromosome position of the duplicated gene. The duplicated gene pairs of the *Triticum aestivum* L. genome were prepared using Circos software [69]. The synteny relationships between the TALE superfamily members in wheat and other species were determined using TBtools [70].

In order to evaluate the selection mode acting on each duplicated *TALE* gene pair, we used TBtools software to calculate the synonymous (Ks) and non-synonymous (Ka) substitution rates between two duplicate *TALE* genes and their respective ratios. Box plots of Ka/Ks ratios were drawn in GraphPad Prism 8 (https://www.graphpad.com/).

### Codon usage pattern analysis

The *TALE* CDS sequences of *Triticum aestivum*, *Oryza sativa, Aegilops tauschii*, *Arabidopsis*, *Solanum tuberosum,* and *Glycine max* were obtained to calculate the codon usage bias by CodonW 1.4.2 software (http://codonw.sourceforge.net/). These parameters included GC3s content, GC content, the codon bias index, and frequency of optimal codons. We also calculated the GC12 content, RSCU, and ENC by EMBOSS tool (https://www.bioinformatics.nl/emboss-explorer/).

### Predicted protein motifs and gene structure characterization of wheat *TALE* genes

Gene structures were visualized using the online drawing tool Gene Structure Display Server 2.0 (http://gsds.cbi.pku.edu.cn/) [71]. The required wheat coding sequence and genomic DNA sequence were downloaded from the Ensembl Plants database. The Multiple Expectation Maximization for Motif Elicitation program (MEME, http://meme-suite.org/tools/meme) [72] was used to examine motifs in the predicted wheat TALE proteins, which were visualized by TBtools. The MEME search parameter was set to a motif count of 20, the motif width ranged from 6 to 200 (inclusive) amino acids, and any number of repetitions were accepted. The detailed amino acid sequences of the 20 motifs are shown in Additional file 12: Table S12.

### Identification of cis-elements in the promoter region of *TALE* genes

The 2000 bp sequence upstream of the start codon of the gene is used as the promoter region [73]. We submitted the promoter region to the PlantCARE database (http://bioinformatics.psb.ugent.be/webtools/plantcare/html/) [74] to identify cis-elements to infer the possible biological function and transcriptional regulation of the *TALE* genes.

### Expression analysis of *TALE* genes in different tissues and abiotic stresses

We obtained data from the wheat expression browser (http://www.wheat-expression.com) [75] to study the expression of wheat *TALE* genes in different tissues and their response to heat and drought. This RNA-seq data comes from nine different growth periods and tissues in Chinese Spring under normal circumstances, and the following six stress and normal control (heat stress for 1 h, drought stress 1 h, heat stress 6 h, drought stress 6 h, combined drought and heat stress 1 h, combined drought and heat stress 6 h, no stress control).

### Interaction network and GO annotation of *TALE* genes in wheat

The TALE protein interaction network was examined using the STRING online server (https://string-db.org/), and visualized with Cytoscape (https://cytoscape.org/).

Blast2GO (https://www.blast2go.com) software was used to perform GO analysis of *TALE* genes in wheat.

### Plant materials, growth conditions, and stress treatments

In this study, seeds of wheat variety “China Spring” were used for hormone and stress treatment. The seeds of wheat (Chinese Spring) was preserved in our experiment. The wheat processing method was done according to previous sources with some modifications [76, 77]. Wheat seeds were grown in an incubator. The temperature of the incubator was 22 °C during the day and 20 °C at night, with a photoperiod of 16 h light/8 h dark. The wheat seeds received hormone and stress treatments 7 days after germination. We placed the seedlings on a dry paper filter for drought stress and in a 42 °C incubator for heat stress. Salt treatment was simulated with 100 mm NaCl solution. Then we exposed the seedlings to 100 μM ABA, 100 μM MeJA, 100 μM ETH, 100 μM GA and other solutions for hormone treatment. Seedling leaves were collected from all treatments and controls at 0, 1, 2, 4, 8, 12, 24 and 48 h. Leaf samples were frozen in liquid nitrogen and stored at − 80 °C.

We used *Arabidopsis* Columbia-0 (Col-0) plants and *knat3* mutant plants for phenotypic analysis to study the function of *TaKNOX11-A*. The growth conditions of *Arabidopsis* were the same as the growth conditions for wheat. We used the *Agrobacterium* infection method to obtain *TaKNOX11-A* transgenic *Arabidopsis*. We ligated the CDS (without termination codon) of *TaKNOX11-A* with the *pCAMBIA1302* vector, and after sequencing verification, transformed it into *Agrobacterium tumefaciens* strain *GV3101*, and transformed *Arabidopsis* Col-0 by the flower soaking method [78]. We used PCR to identify transgenic plants and cultivated the identified plants to the T3 generation. We selected the three lines with the highest expression levels among the T3 generation transgenic lines for stress resistance identification. The seeds of WT and *TaKNOX11-A* overexpression and *knat3* were sterilized and sown on MS medium containing PEG6000 (8 and 10%) (m/v) and NaCl (100 mM and 150 mM) to analyze the germination rate. After 3 days of vernalization, they were moved to an incubator under normal conditions, and the germination rate was counted every 12 h. Seven days after the seeds germinated, seeds with similar germination rates were selected and transferred to MS medium containing 10% PEG6000 or 100 mM NaCl for root length experiments. To evaluate the drought tolerance of the transgenic plants, the 3-week-old seedlings were kept dry for 2 weeks and then watered for 3 days. In order to evaluate the salt tolerance of transgenic plants, seedlings under normal conditions were watered with 100 mM NaCl solution for 7 days, and then watered for 3 days. We performed three independent biological replicates and calculated survival rates.

### RNA extraction and quantitative real-time PCR

We used Trizol to extract RNA from control, stress, and hormone-treated wheat leaves. FastKing RT Kit (TIANGEN, China) was used to remove contaminant DNA and synthesize cDNA. Real-time quantitative PCR was conducted using PerfectStart™ GREEN qPCR SuperMix (SYBR Green I) (Transgen, BEIJING) and QuantStudio 3 real-time PCR system (Thermo Fisher). The wheat β-actin gene was used as an internal reference for all qRT-PCR analyses. We performed four independent replicates for each treatment, and selected the three most reproducible results for subsequent analysis. Gene expression data was analyzed using the 2^-∆∆CT^ value. The qRT-PCR specific primers are listed in Additional File 18: Table S18.

### Physiological index measurement

Test kits (Cominbio, Suzhou, China) were used to measure the pro and MDA content in the leaves of WT, mutant, and transgenic *A. thaliana* seedlings before and after stress. Each measurement was repeated three times.

## Supplementary Information

**Additional file 1: Figure S1.** Syntenic and Evolutionary analyses in wheat TALE family. a) UpSet plot of non-redundant *TALE* genes in different species. b) Violin plot of Ka/Ks rations in duplicated *TALE* gene pairs.

**Additional file 2: Figure S2.** Protein–protein interaction network of wheat TALE proteins.

**Additional file 3: Figure S3.** GO annotation of *TALE* genes in wheat. Biological processes (a), molecular functions (b), and cellular components (c) are annotated.

**Additional file 4: Figure S4.** Germination test of wild-type (WT), *TaKNOX11-A* transgenic *Arabidopsis*, and mutant *Arabidopsis* (*knat3*) seeds under PEG6000 treatment. a) Phenotypes of WT, *TaKNOX11-A* transgenic *Arabidopsis*, and mutant *Arabidopsis* (*knat3*) seeds treated with 0.5 × MS, 8 and 10% PEG6000. b) Germination rates of WT, *TaKNOX11-A* transgenic *Arabidopsis*, and mutant *Arabidopsis* (*knat3*) seeds treated with MS, 8 and 10% PEG6000. Each strain used 36 samples in different treatments for further statistical analysis. Three biological replicates were done for each treatment. The germination rate was counted every 12 h. The error bars indicate the SD of the three replicates.

**Additional file 5: Figure S5.** Germination test of wild-type (WT), *TaKNOX11-A* transgenic *Arabidopsis*, and mutant *Arabidopsis* (*knat3*) seeds under NaCl treatment. a) Phenotypes of WT, *TaKNOX11-A* transgenic *Arabidopsis*, and mutant *Arabidopsis* (*knat3*) seeds treated with 0.5 × MS, and 100 mM and 150 mM NaCl. b) Germination rate of WT, *TaKNOX11-A* transgenic *Arabidopsis*, and mutant *Arabidopsis* (*knat3*) seeds treated with MS, and 100 mM and 150 Mm NaCl. Each strain used 36 samples in different treatments for further statistical analysis. Three biological replicates were done for each treatment. The germination rate was counted every 12 h. The error bars indicate the SD of the three replicates.

**Additional file 6: Figure S6.** The overexpression of *TaKNOX11-A* enhanced drought and salt tolerance in *Arabidopsis*. a) Root length assays of wild-type (WT), *TaKNOX11-A* transgenic *Arabidopsis* and mutant *Arabidopsis* (*knat3*) seeds treated with 10% PEG6000 and 100 mM NaCl. b) Total root lengths of seedlings. c) Fresh weight of normal and stressed *Arabidopsis*. Each strains used three samples in different treatments for further statistical analysis. Three biological replicates were done for each treatment. The error bars indicate the SD of the three replicates. Asterisks indicate significant differences between WT, OE, and mutant lines (*p < 0.05, **p < 0.01, Student’s *t*-test).

**Additional file 7: Figure S7.** The overexpression of *TaKNOX11-A* enhanced drought tolerance in *Arabidopsis*. a) Drought tolerance phenotypes of WT, *TaKNOX11-A* transgenic *Arabidopsis*, and mutant *Arabidopsis* (*knat3*) in soil. Three-week-old seedlings of WT, *TaKNOX11-A* transgenic *Arabidopsis*, and mutant *Arabidopsis* (*knat3*) lines were dehydrated for 1 week and then rehydrated for 3 days. b) Survival rate of normal and drought-stressed *Arabidopsis*. c-d) Proline and malondialdehyde content were detected in WT, OE, and mutant plants under normal growth and drought conditions. Twelve samples per strains were used for the drought treatment and for further statistical analysis. Three biological replicates were done for each treatment. Data were presented as the mean ± SD of three independent replicates. Asterisks indicate significant differences between WT, OE, and mutant lines (*p < 0.05, ** *p* < 0.01, Student’s *t*-test).

**Additional file 8: Figure S8.** The overexpression of *TaKNOX11-A* enhanced salt tolerance in *Arabidopsis*. Mutant *Arabidopsis* (*knat3*) showed lower salt resistance compared to wild-type (WT) plants. a) NaCl tolerance phenotypes of WT, *TaKNOX11-A* transgenic *Arabidopsis*, and mutant *Arabidopsis* (*knat3*) in soil. Three-week-old seedlings of WT, *TaKNOX11-A* transgenic *Arabidopsis*, and mutant *Arabidopsis* (*knat3*) lines were salt stressed for 1 week and then rewatered for 3 days. b) Survival rate of normal and salt-stressed *Arabidopsis*. c-d) Proline and malondialdehyde content were detected in WT, OE, and mutant plants under normal growth and salt stress conditions. Twelve samples per strains were used for the salt stress treatment and for further statistical analysis. Three biological replicates were done for each treatment. Data are presented as the mean ± SD of three independent replicates. Asterisks indicate significant differences between WT, OE, and mutant lines (*p < 0.05, ** *p* < 0.01, Student’s *t*-test).

**Additional file 9: Table S9.** The basic information of 70 wheat *TALE* genes.

**Additional file 10: Table S10.** Homoeologous *TALE* genes in wheat.

**Additional file 11: Table S11.** The Ka/Ks ratios of duplicated *TALE* gene pairs in wheat and other plants.

**Additional file 12: Table S12**. List of the 20 identified motifs in wheat TALE proteins.

**Additional file 13: Table S13.** Cis-acting elements of 70 wheat *TALE* gene promoter regions.

**Additional file 14: Table S14.** The tpm values of wheat *TALE* genes in the different wheat developmental stages and stress treatments.

**Additional file 15: Table S15.** GO annotation of *TALE* genes in wheat. The annotation was performed on three categories, (a) biological process, (b) molecular function and (c) cellular component.

**Additional file 16: Table S16.** The germination rates of WT, *TaKNOX11-A* transgenic *Arabidopsis*, and mutant *Arabidopsis* (*knat3*) seeds under PEG6000 and NaCl treatments.

**Additional file 17: Table S17.** Measured values for root length, fresh weight, and content of proline and malondialdehyde.

**Additional file 18: Table S18.** List of primers used in this study.

## Data Availability

All data generated or analyzed during this study are included within the article and its additional files. Protein sequences of different species are obtained from the Ensembl Plants database (http://plants.ensembl.org/index.html), NCBI (https://www.ncbi.nlm.n-ih.gov/), and TAIR (https://www.arabidopsis.org/). The RNA-Seq data involved in this study downloaded from the Wheat Expression Browser (http://www.wheat-expression.com).

## Abbreviations

TALE: Three-amino-loop-extension protein
KNOX: KNOTTED-like homeodomain
BELL: BEL1-like homeodomain
CUB: Codon Usage Bais
ENC: Effective number of codon
RSCU: Relative Synonymous Codon Usage
MeJA: Methyl jasmonate
ABA: Abscisic Acid
ETH: Ethylene
GA: Gibberellin
MS: Murashige and Skoog
Ka: Non-synonymous
Ks: Synonymous
Pro: Proline
MDA: Malondialdehyde
NES: Nuclear export signal
PEG6000: Polyethylene glycol 6000
Mw: Molecular weight
pI: Isoelectric point
SD: Standard deviation
TPM: Transcripts PerKilobase Million
