# Supplementary figures and images for: Genome-wide analysis of TALE superfamily in Triticum aestivum reveals TaKNOX11-A is involved in abiotic stress response
Source: BMC Genomics. 2022 Jan 31;23:89. doi: 10.1186/s12864-022-08324-y (PMC8805372; doi:10.1186/s12864-022-08324-y)

a

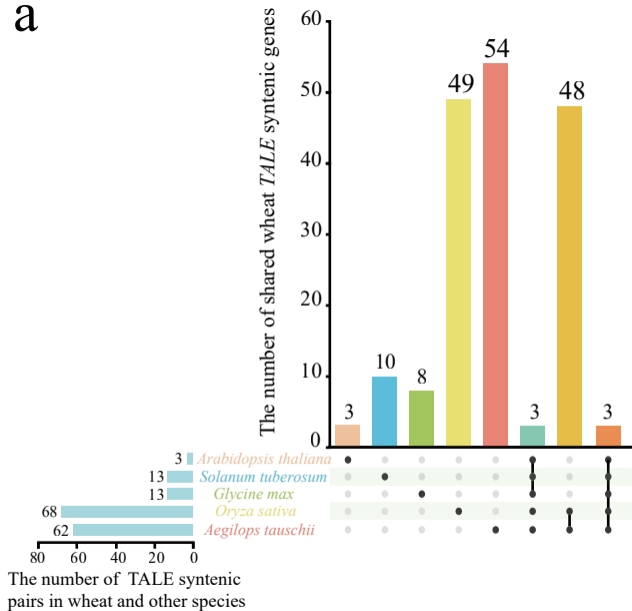

b

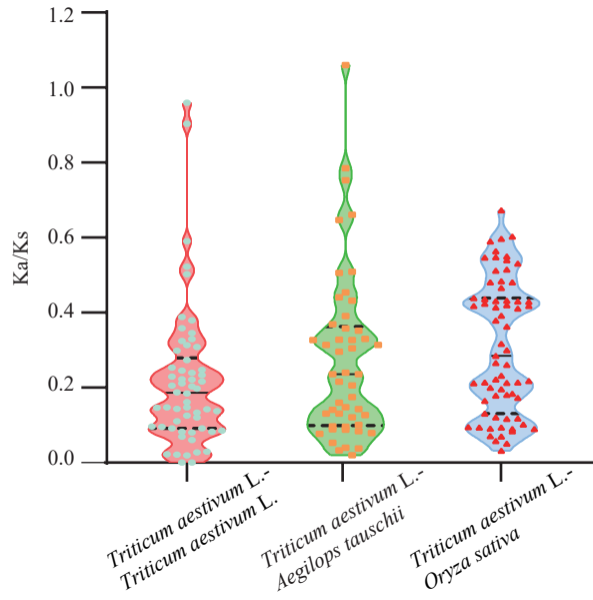

Supplement: Supplementary file 1 — Additional file 1: Figure S1. Syntenic and Evolutionary analyses in wheat TALE family. a) UpSet plot of non-redundant TALE genes in different species. b) Violin plot of Ka/Ks rations in duplicated TALE gene pairs. [file 12864_2022_8324_MOESM1_ESM.pdf]

## GO terms

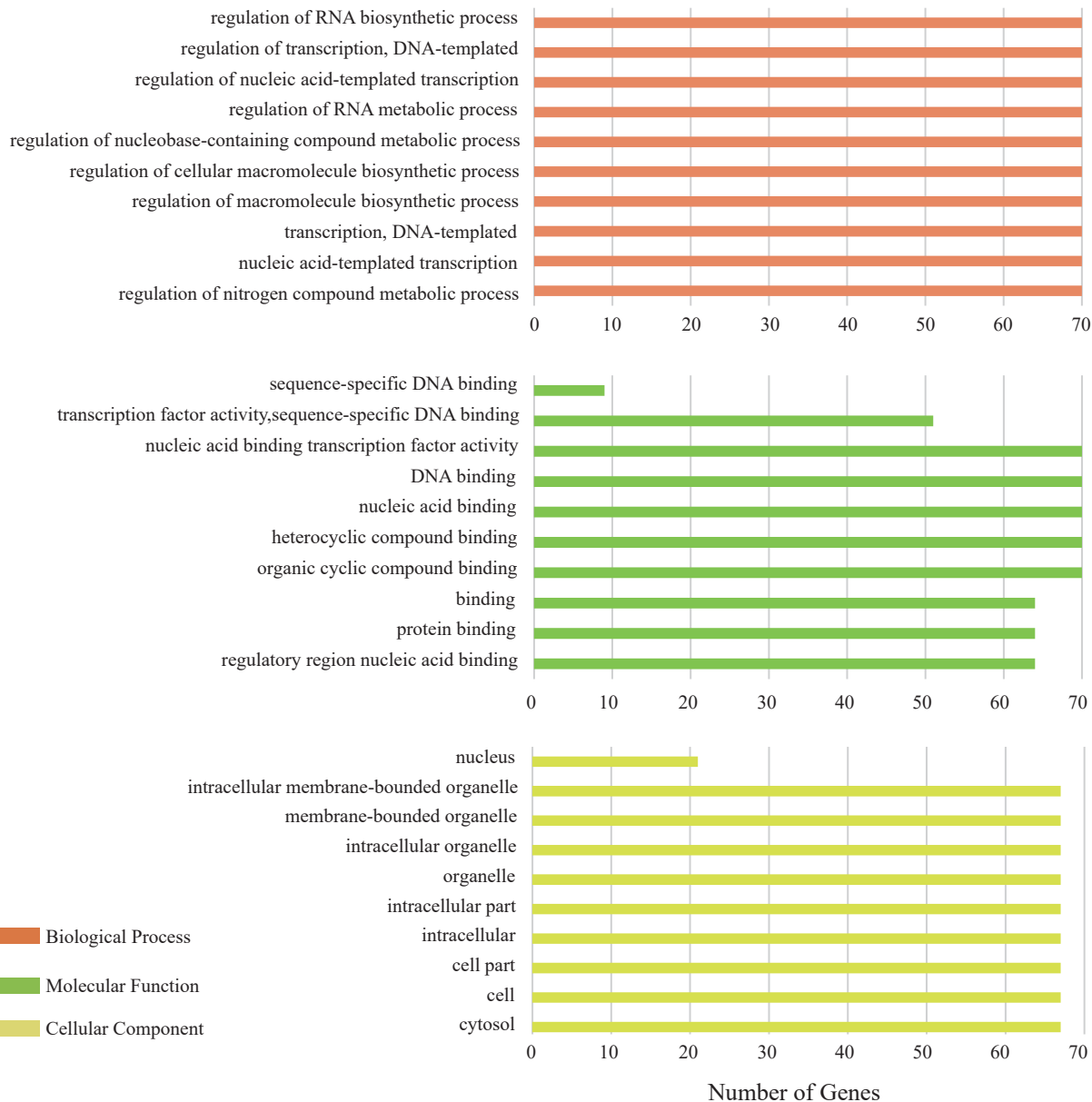

Supplement: Supplementary file 3 — Additional file 3: Figure S3. GO annotation of TALE genes in wheat. Biological processes (a), molecular functions (b), and cellular components (c) are annotated. [file 12864_2022_8324_MOESM3_ESM.pdf]

a

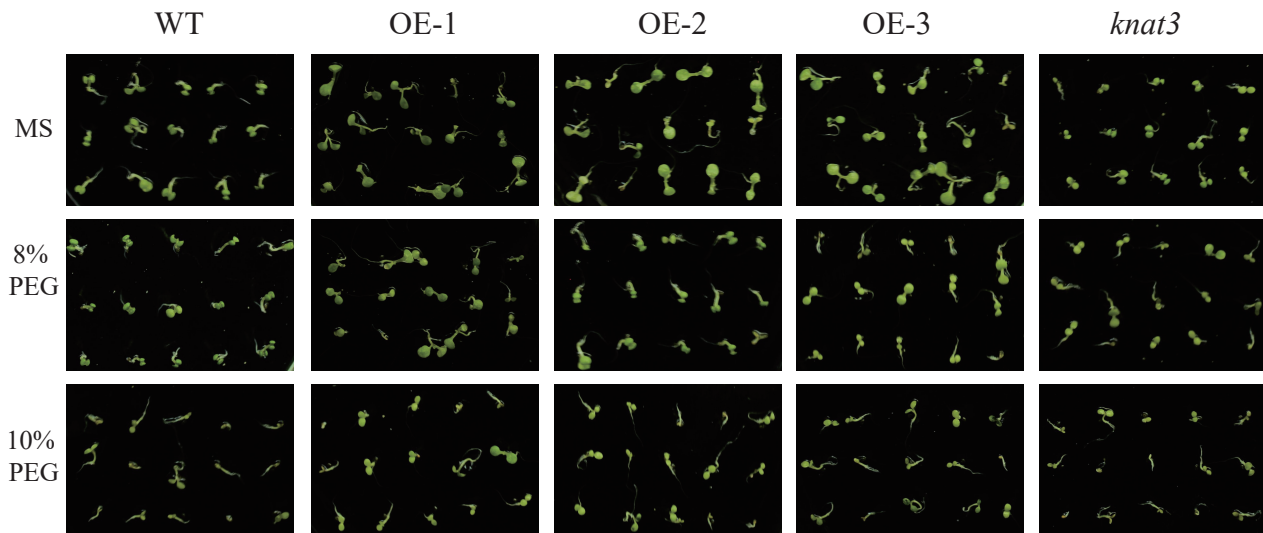

b

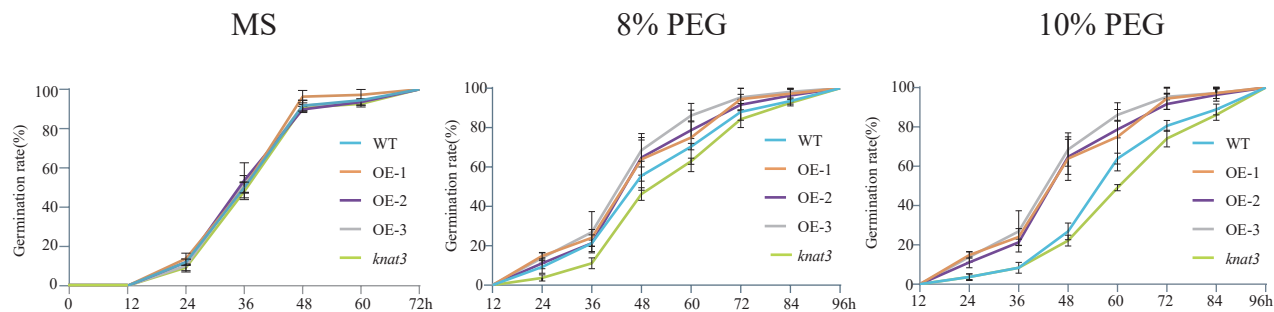

Supplement: Supplementary file 4 — Additional file 4: Figure S4. Germination test of wild-type (WT), TaKNOX11-A transgenic Arabidopsis, and mutant Arabidopsis (knat3) seeds under PEG6000 treatment. a) Phenotypes of WT, TaKNOX11-A transgenic Arabidopsis, and mutant Arabidopsis (knat3) seeds treated with 0.5 × MS, 8 and 10% PEG6000. b) Germination rates of WT, TaKNOX11-A transgenic Arabidopsis, and mutant Arabidopsis (knat3) seeds treated with MS, 8 and 10% PEG6000. Each strain used 36 samples in different treatments for further statistical analysis. Three biological replicates were done for each treatment. The germination rate was counted every 12 h. The error bars indicate the SD of the three replicates. [file 12864_2022_8324_MOESM4_ESM.pdf]

a

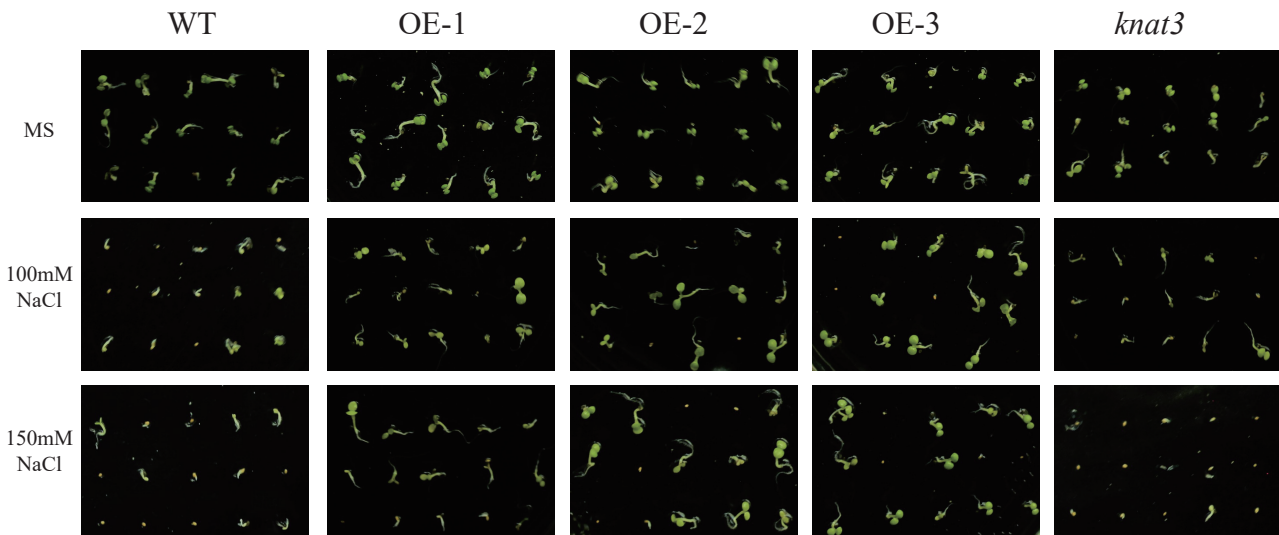

b

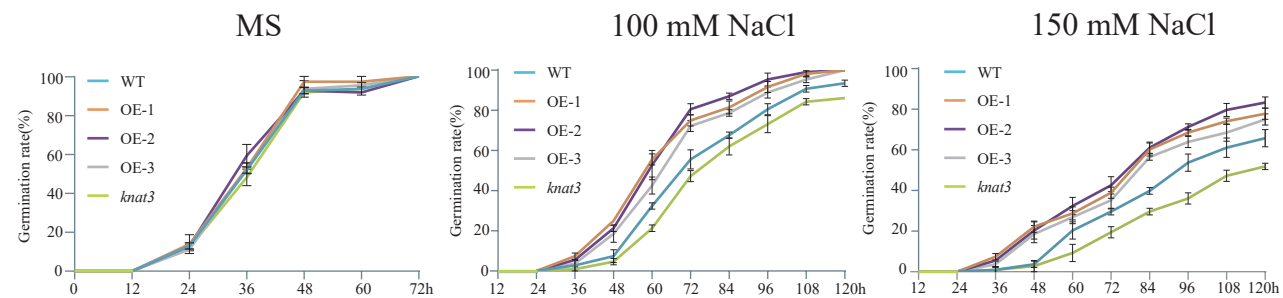

Supplement: Supplementary file 5 — Additional file 5: Figure S5. Germination test of wild-type (WT), TaKNOX11-A transgenic Arabidopsis, and mutant Arabidopsis (knat3) seeds under NaCl treatment. a) Phenotypes of WT, TaKNOX11-A transgenic Arabidopsis, and mutant Arabidopsis (knat3) seeds treated with 0.5 × MS, and 100 mM and 150 mM NaCl. b) Germination rate of WT, TaKNOX11-A transgenic Arabidopsis, and mutant Arabidopsis (knat3) seeds treated with MS, and 100 mM and 150 Mm NaCl. Each strain used 36 samples in different treatments for further statistical analysis. Three biological replicates were done for each treatment. The germination rate was counted every 12 h. The error bars indicate the SD of the three replicates. [file 12864_2022_8324_MOESM5_ESM.pdf]

a

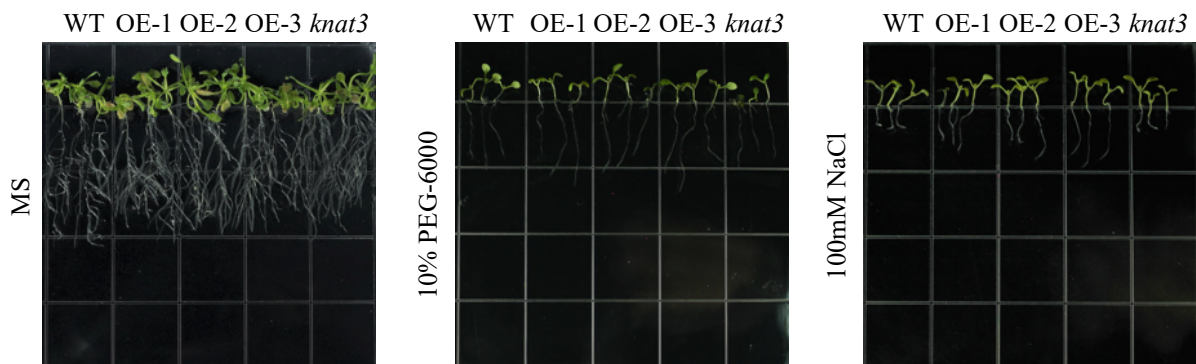

b

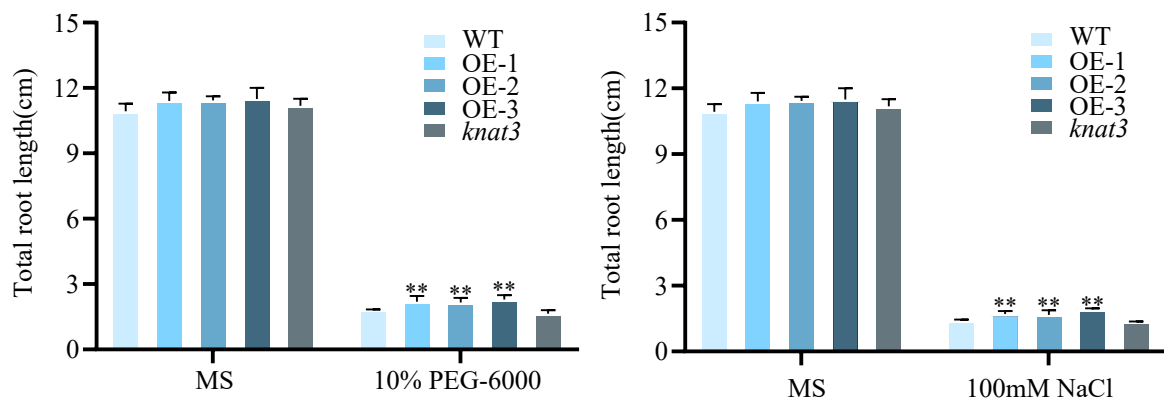

c

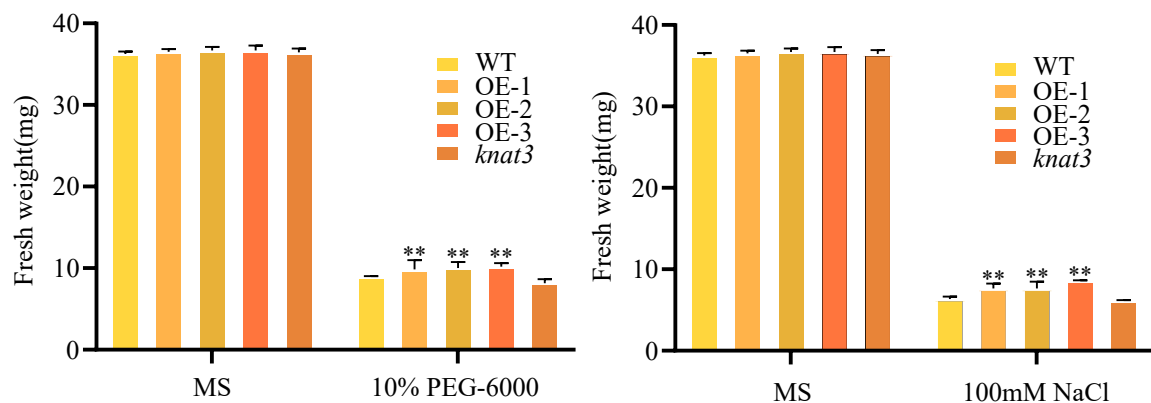

Supplement: Supplementary file 6 — Additional file 6: Figure S6. The overexpression of TaKNOX11-A enhanced drought and salt tolerance in Arabidopsis. a) Root length assays of wild-type (WT), TaKNOX11-A transgenic Arabidopsis and mutant Arabidopsis (knat3) seeds treated with 10% PEG6000 and 100 mM NaCl. b) Total root lengths of seedlings. c) Fresh weight of normal and stressed Arabidopsis. Each strains used three samples in different treatments for further statistical analysis. Three biological replicates were done for each treatment. The error bars indicate the SD of the three replicates. Asterisks indicate significant differences between WT, OE, and mutant lines (*p < 0.05, **p < 0.01, Student’s t-test). [file 12864_2022_8324_MOESM6_ESM.pdf]

**a**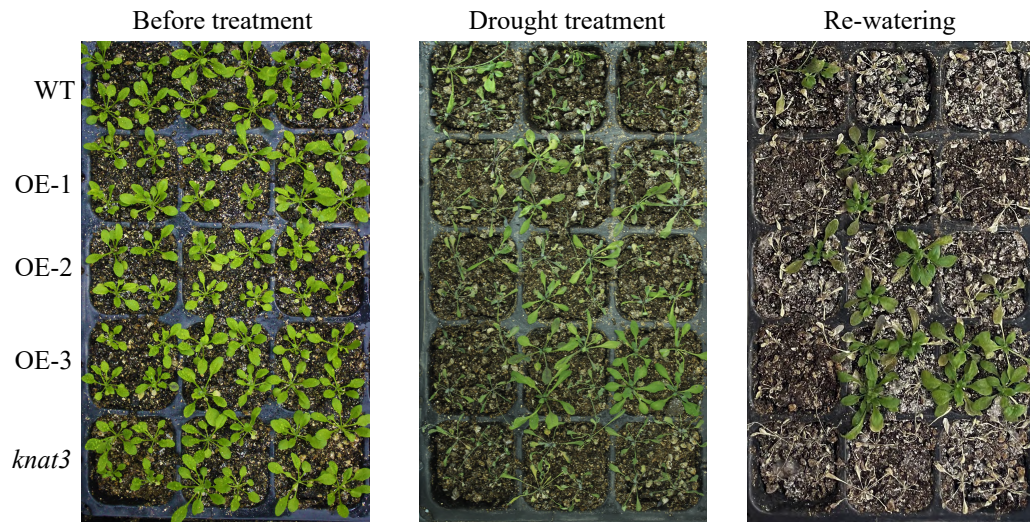**b**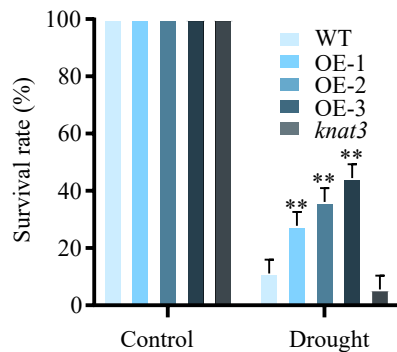**c**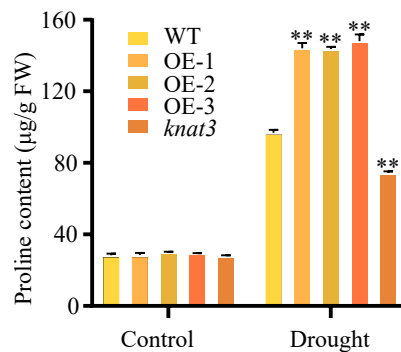**d**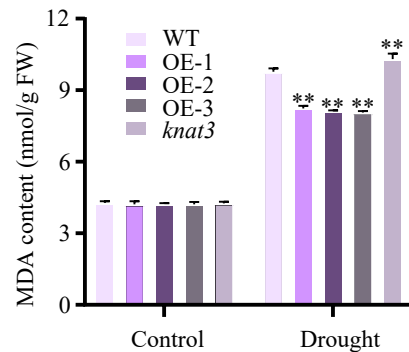

Supplement: Supplementary file 7 — Additional file 7: Figure S7. The overexpression of TaKNOX11-A enhanced drought tolerance in Arabidopsis. a) Drought tolerance phenotypes of WT, TaKNOX11-A transgenic Arabidopsis, and mutant Arabidopsis (knat3) in soil. Three-week-old seedlings of WT, TaKNOX11-A transgenic Arabidopsis, and mutant Arabidopsis (knat3) lines were dehydrated for 1 week and then rehydrated for 3 days. b) Survival rate of normal and drought-stressed Arabidopsis. c-d) Proline and malondialdehyde content were detected in WT, OE, and mutant plants under normal growth and drought conditions. Twelve samples per strains were used for the drought treatment and for further statistical analysis. Three biological replicates were done for each treatment. Data were presented as the mean ± SD of three independent replicates. Asterisks indicate significant differences between WT, OE, and mutant lines (*p < 0.05, ** p < 0.01, Student’s t-test). [file 12864_2022_8324_MOESM7_ESM.pdf]

a

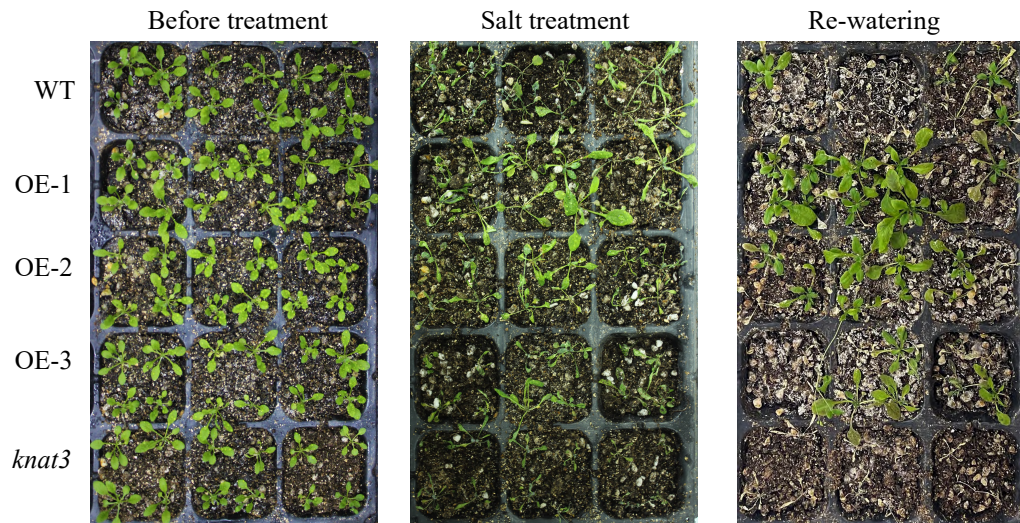

b

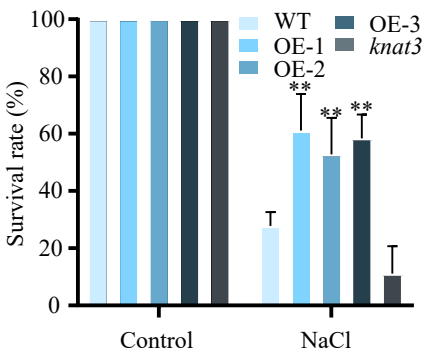

c

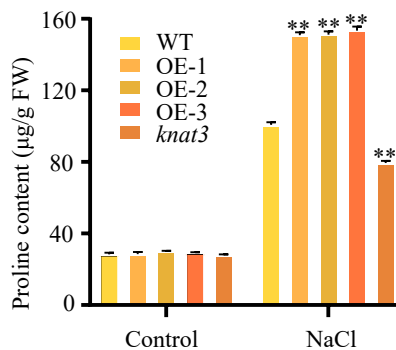

d

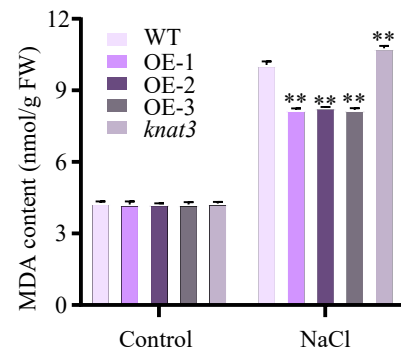

Supplement: Supplementary file 8 — Additional file 8: Figure S8. The overexpression of TaKNOX11-A enhanced salt tolerance in Arabidopsis. Mutant Arabidopsis (knat3) showed lower salt resistance compared to wild-type (WT) plants. a) NaCl tolerance phenotypes of WT, TaKNOX11-A transgenic Arabidopsis, and mutant Arabidopsis (knat3) in soil. Three-week-old seedlings of WT, TaKNOX11-A transgenic Arabidopsis, and mutant Arabidopsis (knat3) lines were salt stressed for 1 week and then rewatered for 3 days. b) Survival rate of normal and salt-stressed Arabidopsis. c-d) Proline and malondialdehyde content were detected in WT, OE, and mutant plants under normal growth and salt stress conditions. Twelve samples per strains were used for the salt stress treatment and for further statistical analysis. Three biological replicates were done for each treatment. Data are presented as the mean ± SD of three independent replicates. Asterisks indicate significant differences between WT, OE, and mutant lines (*p < 0.05, ** p < 0.01, Student’s t-test). [file 12864_2022_8324_MOESM8_ESM.pdf]
