# Supplementary material for: Genome-wide analysis of TALE superfamily in Triticum aestivum reveals TaKNOX11-A is involved in abiotic stress response
Source: BMC Genomics. 2022 Jan 31;23:89. doi: 10.1186/s12864-022-08324-y (PMC8805372; doi:10.1186/s12864-022-08324-y)
Supplement: Supplementary file 2 — Additional file 2: Figure S2. Protein–protein interaction network of wheat TALE proteins. [file 12864_2022_8324_MOESM2_ESM.pdf]

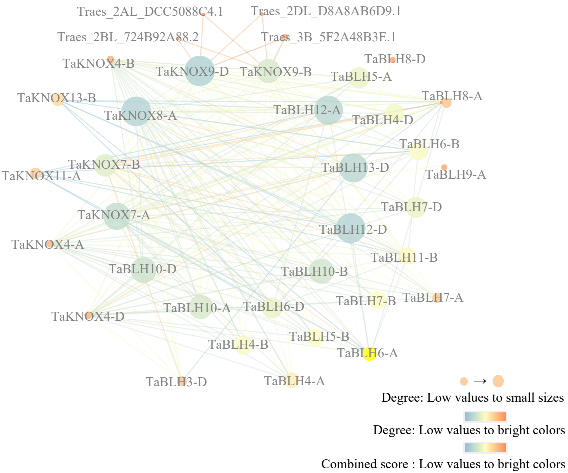

Traes_2AL_DCC5088C4.1
Traes_2DL_D8A8AB6D9.1
Traes_2BL_724B92A88.2
Traes_3B_5F2A48B3E.1
TaKNOX4-B
TaKNOX9-D
TaKNOX9-B
TaBLH8-D
TaBLH5-A
TaKNOX13-B
TaBLH8-A
TaKNOX8-A
TaBLH12-A
TaBLH4-D
TaBLH6-B
TaKNOX7-B
TaKNOX11-A
TaBLH13-D
TaBLH9-A
TaBLH7-D
TaKNOX7-A
TaBLH12-D
TaKNOX4-A
TaBLH11-B
TaBLH10-D
TaBLH10-B
TaBLH7-B
TaBLH7-A
TaKNOX4-D
TaBLH10-A
TaBLH6-D
TaBLH5-B
TaBLH4-B
TaBLH6-A
TaBLH3-D
TaBLH4-A
Degree: Low values to small sizes
Degree: Low values to bright colors
Combined score : Low values to bright colors
